# Differential Functional Constraints on the Evolution of Postsynaptic Density Proteins in Neocortical Laminae

**DOI:** 10.1371/journal.pone.0039686

**Published:** 2012-06-28

**Authors:** Guang-Zhong Wang, Genevieve Konopka

**Affiliations:** Department of Neuroscience, The University of Texas at Southwestern Medical Center, Dallas, Texas, United States of America; University of California, San Diego, United States of America

## Abstract

The postsynaptic density (PSD) is a protein dense complex on the postsynaptic membrane of excitatory synapses that is implicated in normal nervous system functions such as synaptic plasticity, and also contains an enrichment of proteins involved in neuropsychiatric disorders. It has recently been reported that the genes encoding PSD proteins evolved more slowly than other genes in the human brain, but the underlying evolutionary advantage for this is not clear. Here, we show that cortical gene expression levels could explain most of this effect, indicating that expression level is a primary contributor to the evolution of these genes in the brain. Furthermore, we identify a positive correlation between the expression of PSD genes and cortical layers, with PSD genes being more highly expressed in deep layers, likely as a result of layer-enriched transcription factors. As the cortical layers of the mammalian brain have distinct functions and anatomical projections, our results indicate that the emergence of the unique six-layered mammalian cortex may have provided differential functional constraints on the evolution of PSD genes. More superficial cortical layers contain PSD genes with less constraint and these layers are primarily involved in intracortical projections, connections that may be particularly important for evolved cognitive functions. Therefore, the differential expression and evolutionary constraint of PSD genes in neocortical laminae may be critical not only for neocortical architecture but the cognitive functions that are dependent on this structure.

## Introduction

The postsynaptic density (PSD) is a unique complex of the excitatory synapse containing hundreds of proteins, many of which are critical for complex neurological processes such as synaptic plasticity [1]–[3]. Many of the proteins in the PSD complex are neurotransmitter receptors that are important for signal processing as well as normal cognitive functions [2], [4]. In addition, recent work has demonstrated that human PSD genes, along with many other genes, are implicated in over 100 neurological and psychiatric diseases, and these genes evolved more slowly not only when compared to the other genes in the genome, but also when compared to other brain-related genes [5]. The conservation of these genes in the brain indicates that there are more evolutionary constraints on the sequences of these genes compared to other brain-related genes, but the underlying functional impetus for this finding is not fully understood.

Other recent work has shown that newly evolved genes, or young genes, which are defined as genes that are specifically expressed in the primate lineage, are significantly enriched in the human fetal neocortex [6]. The recruitment of young genes into human neocortex suggests a link between the evolution of the genes and the function of the tissue. Moreover, genes in the brain are usually nonuniformly expressed, with specific patterns of gene expression in distinct areas of the brain that not only include large regional differences [7], but also differences in more neuroanatomically refined areas such the neocortical layers [8]. Taking this idea one step further, a recent study has shown that there are subregional differences in gene expression among different strains of mice [9], indicating that genetic variation drives additional variation in gene expression. However, the evolutionary importance of these expression patterns also remains unknown.

The six-layered cortex is one of the hallmarks of mammalian brain evolution; not only is the cortex the most recently evolved structure in the brain, but its development was likely critical for the emergence of higher cognition [10]–[12]. Understanding the function of these layers through examination at many levels from gene expression through circuitry is expected to provide insight into cognition [8], [13]. Since there is an enrichment of PSD proteins that are involved in neuropsychiatric disorders [5], we hypothesized that expression patterns in the cortex may provide clues to the evolution of PSD proteins. Here, we analyze the relationship between PSD gene evolution and the architecture of neocortical laminae in the mouse and rhesus macaque cortex (somatosensory and visual cortices). We find that the structure of the six-layered cortex provided functional constraints on the evolution of PSD genes. Moreover, the pattern of functional constraint – superficial layers have less constraint than deep layers – supports a potential role for PSD protein involvement in cognition since cortico-cortical connections may have been important for the evolution of higher-order cognitive learning.

## Results

We first determined whether PSD genes in mouse brain indeed show slower evolutionary rates than other brain related genes. Therefore, we obtained the mouse orthologs of recently identified human PSD genes and mapped the evolutionary parameters to these orthologs [5]. The principle findings reported here were also found using a mouse PSD gene set which has a smaller sample size [14]. We confirmed that PSD genes have lower evolutionary rates (dN/dS) than all other genes in the mouse genome (mean dN/dS values for PSD genes: 0.0654+/−0.0021, mean dN/dS values for non-PSD genes: 0.1151+/−0.0012, *p*<2.2e–16, Wilcoxon rank sum test). This is expected as tissue-specific genes typically have different evolutionary rates, and brain-related genes have lower evolutionary rates than other tissue-specific genes [15], [16]. Therefore, we asked whether the evolutionary rate of PSD genes was different than that of other non-PSD brain-related genes. Again as previously shown [5], PSD genes have a lower evolutionary rate compared to seven different brain related gene categories (see Materials and Methods; Table 1).

**Table 1:** Comparison of the average evolutionary rate (dN/dS) of PSD proteins.

	Before controlling for expression level			After controlling for expression level				
Categories	Other brain related genes	PSD genes	*p*		Other brain related genes	PSD genes	*p*	
**Layers 2/3**	0.1156+/−0.0026	0.0660+/−0.0023	<2.2×10^−16^		0.00099+/−0.00248	−0.01136+/−0.00244	0.2	
**Layer 4**	0.1205+/−0.0065	0.0658+/−0.0021	<2.2×10^−16^		−0.01665+/−0.00620	−0.01096+/−0.0022	1.00	
**Layer 5**	0.1180+/−0.0023	0.0667+/−0.0027	<2.2×10^−16^		0.00995+/−0.00221	−0.01192+/−0.00284	1.3×10^−5^	
**Layer 6**	0.1129+/−0.0053	0.0646+/−0.0021	<2.2×10^−16^		−0.00406+/−0.00504	−0.01276+/−0.00219	0.3	
**Layer 6b**	0.1128+/−0.0036	0.0653+/−0.0022	<2.2×10^−16^		−0.00173+/−0.00358	−0.0118+/−0.00229	0.26	
**Mouse brain proteomics**	0.0938+/−0.0018	0.0648+/−0.0025	<2.2×10^−16^		−0.00277+/−0.00184	−0.01021+/−0.00262	0.15	
**Mouse brain plasma membrane proteomics**	0.0884+/−0.0056	0.0657+/−0.0022	7×10^−5^		−0.00058+/−0.00584	−0.01154+/−0.00231	0.121	

Orthologous mouse proteins were compared with other brain related proteins before and after controlling for mean expression levels across mouse cortical layers. *p* values were calculated by a one tailed Wilcoxon rank sum test upon comparing enriched genes in each category to PSD genes overall.

**Figure 1:**
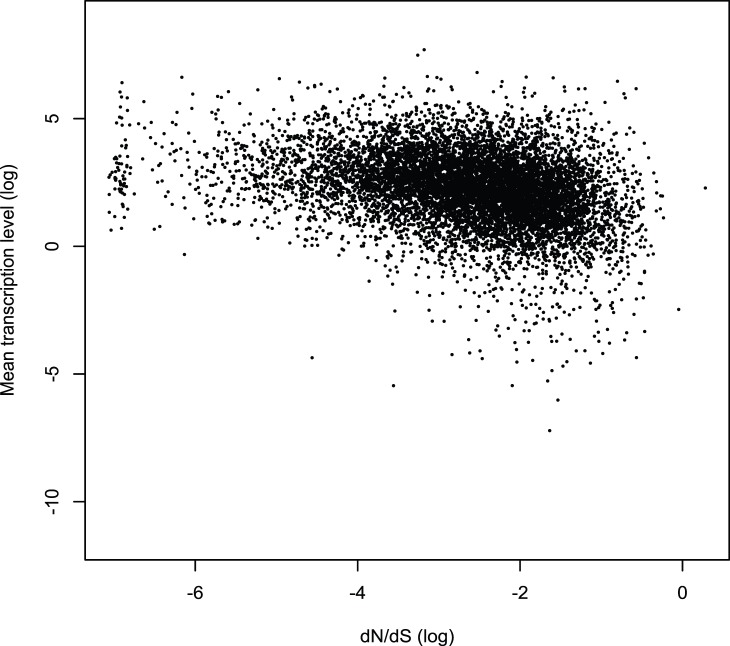
Mean transcription levels in mouse cortex and the evolutionary rates (dN/dS) of these genes are negatively correlated (Spearman's *r*  = −**0.34,** ***p*** **<2.2×10** ^−**16**^ **).**

Many genomic factors affect the evolutionary rate of proteins, such as recombination rate, gene dispensability, network neighbors, number of protein interactions and expression level [17]–[19]. For example, gene expression levels can explain half of the variation in the evolution rate of yeast proteins [20], and the effect of gene expression on protein evolution has been extended to other species such as human, where it may be important for reducing the cost of protein misfolding [21]. Indeed, we found that there is a negative correlation between transcriptional abundance of mouse cortical genes and their evolutionary rate (Spearman's *r*  =  −0.34, *p*<2.2e–16, Figure 1). To determine whether gene expression levels in the cortex are driving the low evolutionary rate of PSD genes compared to other brain related genes, we used mouse cortical gene expression data to control for the effects of gene expression [8]. Although PSD genes continue to show significantly lower evolutionary rates than other proteins in the genome after controlling for transcription level in the genome (mean dN/dS values for PSD genes: −0.0117+/−0.0022, mean dN/dS values for non-PSD genes: 0.0047+/−0.0011, *p*  = 0.0015,Wilcoxon rank sum test), this relationship does not hold for most of the brain-expressed genes datasets we compared (Table 1). These results indicate that the low evolutionary rate of PSD genes compared with other brain related genes could primarily be explained by transcriptional abundance in the cortex, suggesting that the transcriptional level of a gene is a substantial contributing factor to constraining the evolution of these genes in the brain.

**Figure 2:**
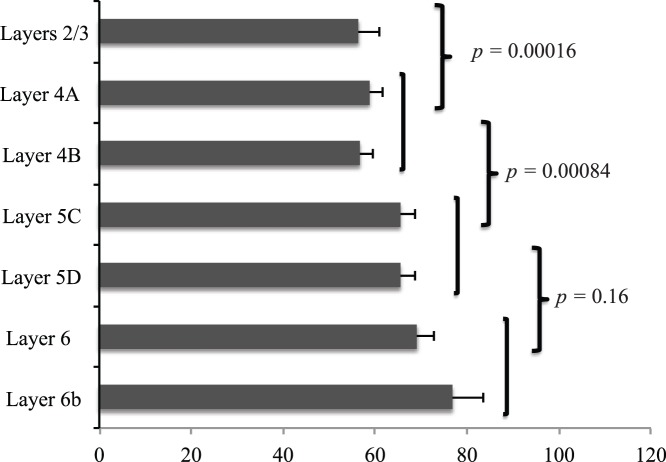
Comparison of the expression level of PSD genes in different layers of the mouse cortex. PSD genes are more highly expressed in deep layers in the mouse cortex. *p* values were calculated by a Wilcoxon rank sum test.

We next determined whether constraint on PSD gene evolution was related to the evolution of a six-layered cortex. Therefore, we examined whether the layers of the cortex express different amounts of PSD genes by cross-referencing a genome-wide transcriptional atlas of mouse cortical layers (somatosensory cortex) [8] to examine layer-specific expression of 1230 PSD genes. Consistent with transcriptional level being a major contributing factor to the low evolutionary rate of PSD genes, the mean transcriptional abundance of the PSD genes in each layer is higher than all of the other genes in each layer (*p*<2×10^−15^ in all of the comparisons, Wilcoxon rank sum test). Interestingly, the layers express an increasing amount of PSD genes with increasing depth of the layers (Figure 2), indicating that cortical layers have differential evolutionary constraints on PSD genes with deeper layers having more constraint than upper layers. Moreover, upon examination of evolutionary rates of PSD proteins in each layer, we found that PSD genes in general have significantly lower rates than non-PSD genes enriched in layer 5 (Table 1). Conducting similar analyses using genes encoding for presynaptic proteins (see Methods), we see a similar trend for genes in deeper layers having greater expression (Table S1). Since extensive profiling and large-scale validation of presynaptic proteins has not been conducted in human tissue, we will limit our discussion to PSD genes. However, it is possible that many of our findings may be relevant to the synapse in general, instead of only the postsynaptic side.

Using recently available rhesus macaque neocortical layer data [22], we also observed that PSD genes in deep layers (layers 5–6) have significantly higher expression levels than those in upper layers (layers 1–3) in both the primary visual cortex (V1) and secondary visual cortex (V2) (8.50+/−0.071 vs. 8.45+/−0.068, *p*  = 2×10^−4^ for primary visual cortex (V1) and 8.49+/−0.071 vs. 8.44+/−0.069, *p*  = 7.4×10^−6^ for secondary visual cortex (V2); significance levels were determined by a paired Wilcoxon signed rank test; Figure S1). Thus, this observed difference in PSD gene expression between upper and lower cortical layers is likely applicable to all mammals. To rule out the possibility that a few highly expressed PSD genes were driving these results, we plotted the density of PSD genes in each layer as a function of mean transcriptional level for both the mouse and macaque expression data (Figures S2 and S3). We find similar numbers of highly expressed PSD genes in each layer, supporting the validity of our results.

Next, we examined whether there is an enrichment of the number of PSD genes transcribed in specific layers. By mapping PSD genes to the genes differentially expressed in each layer [8], we found that more than one-third of PSD genes are preferentially located in layer 5 (*p*  = 9.01e–08, Fisher's exact test) (Table 2). This result cannot be explained by increased neuronal density in layer 5, which has not been found in mouse or rat somatosensory cortex nor human temporal or anterior cingulate cortex [23], [24]. In fact, in a study of NeuN positive neurons in the rat somatosensory cortex, layer 5 had fewer neurons than layers 2, 3, or 6 [24]. Furthermore, other layers do not show significant enrichment of PSD genes (Table 2). We also controlled for cellular density throughout the cortex by normalizing our PSD expression data to an abundant specific marker of astrocytes, *Aldh1l1* [25], [26]. We find the same increase in PSD gene expression in deeper layers of the cortex after normalization (Table S2). These findings, together with the discovery that nearly half of Parkinson’s disease-related genes are enriched in layer 5 [8], highlights the potentially unique role of layer 5 in neurological diseases.

**Table 2:** PSD genes are enriched in layer 5-enriched genes.

	PSD	Non-PSD	*p* value
**Layers 2/3 enriched**	232	2371	1.00
**Layer 4 enriched**	23	706	1.00
**Layer 5 enriched**	416	2710	9×10^−8^
**Layer 6 enriched**	47	571	1.00
**Layer 6b enriched**	123	982	0.36
**Total PSD**	1230		
**Total non-PSD**		10180	

*p* values from a one tailed Fisher’s exact test are shown.

Finally, we investigated the mechanism by which PSD genes are being highly transcribed in deeper layers. One potential mechanism is that these genes tend to be transcribed by layer-enriched transcription factors. Due to the lack of genome wide transcription factor and target gene relationships in mammalian genomes, we generated a large-scale transcription factor and PSD gene co-expression dataset (See Materials and Methods) [27]. Based on our hypothesis, two predictions could be made. First, layer-enriched PSD genes themselves should have higher co-expression levels in deep layers. Second, there should be higher co-expression levels between PSD proteins and transcription factors that prefer to be expressed in deep layers. To test the first possibility, we mapped all of the PSD genes to the layer-enriched genes, and calculated the co-expression levels between each gene pair. We found that PSD genes have higher co-expression levels than other gene pairs in the co-expression network (mean co-expression level: 0.165+/−0.001 *vs.* 0.120+/−0.006, *p*  = 6×10^−12^), consistent with the fact that PSD genes are highly connected in the synapse [2], [28]. Layer 5-enriched PSD genes have the highest co-expression level than any other layer-enriched PSD gene group (*p*<0.00001 in all of the comparisons), indicating that layer 5 PSD proteins are more likely to be functionally coordinated. This is further evidence for the critical role of PSD proteins in layer 5 of the cortex. The co-expression of PSD gene pairs in layers 6 is also higher than the co-expression of PSD gene pairs in layer 2/3 or 4 (*p*<0.0001 in both of the comparisons). Additionally, the co-expression of PSD gene pairs in deep layers still have significantly higher co-expression levels than the co-expression of genes in upper layers if only the top 50% highly expressed or bottom 50% expressed PSD genes are used (*p*<0.001 in all of the comparisons; Table S3), which rules out the possibility that decreased expression could lead to lower co-expression values. To test our second prediction, we examined the co-expression relationships between PSD genes and transcription factors [29]. PSD genes have higher co-expression values with layer-specific transcription factors than other genes (mean co-expression level: 0.125+/−0.001 *vs.* 0.079+/−0.006, *p*  = 6.7×10^−15^), indicating that these genes are more likely to be regulated by layer-enriched transcription factors. In addition, we find that the co-expression of layer 6-enriched transcription factors and PSD genes is higher than between any of the other layers (*p*<0.0001 in all of comparisons), and the co-expression levels of enriched transcription factors in layer 5 and layer 4 with PSD genes are higher than that of layer 2/3 (*p*<10^−12^ in both cases, and is also true if we only include the top 50% or bottom 50% expressed PSD genes, Table S4) (see Materials and Methods).

## Discussion

The role of tissue specificity on functional constraint in the evolution of genes is a largely unexplored topic in the molecular evolution field, especially in the nervous system. Recent work has found an enrichment of new genes in the human neocortex [6], suggesting that the evolution of the cortex required new functional pathways and properties for its enhanced functions. Moreover, there are thousands of genes showing patterned expression across different neocortical layers indicating a potential role for the neocortex on the evolution of brain related genes [8]. Therefore, we examined whether this regional tissue-specificity within the neocortex is correlated with the evolution of genes expressed in the cortex. In addition, we focused on genes encoding for PSD proteins since these proteins have been strongly implicated in neuropsychiatric disorders [5], [30], [31].

Previous work demonstrated that PSD genes are significantly constrained compared to other brain-related genes [5]. Our results suggest that the low evolutionary rate of PSD genes can be explained by the transcriptional abundance of these genes when using mouse somatosensory cortex transcriptome data. The exception to this finding is the rate of genes in layer 5, as discussed below. It is also possible that genes expressed outside of the cortex have significantly lower evolutionary rates after accounting for expressing levels. This possibility should be explored in future studies. However, given the emergence of a six-layered cortex in mammals [10]–[12], overall relaxed constraint of cortical PSD genes would be beneficial for the evolution of this tissue.

We also observe that PSD genes are enriched and more highly transcribed in deeper layers. However, the graded increase in PSD gene expression from layers 2/3 to layer 4, for example, does not correlate with an increase in the proportion of PSD genes from layers 2/3 (10%) to layer 4 (3%). Thus, it is possible that lower PSD expression in layers 2/3 overall is offset by a greater number of functional or more efficient PSD proteins. Interestingly, layer 5 has the greatest number of PSD genes with enriched expression (Table 2) and the evolutionary rate of PSD genes in general is significantly less than non-PSD genes in layer 5 after controlling for expression amounts (Table 1). In addition, it was previously found that layer 5 neurons are enriched for genes involved in Parkinson’s disease [8]. While functional recordings of cortical neurons have uncovered spontaneous activity in neurons of layers 5 and 6 [32], [33], layer 5 neurons appear to have an enhanced excitability to propagate electrical activity within layer 5 forming a tightly coupled circuit within the layer [32], [34]. Moreover, layer 5 neurons are the only neurons in the cortex that project to the spinal cord, midbrain, and hindbrain [35]. Interestingly, layer 5 neurons have the greatest number of dendritic spines compared to other pyramidal neurons in the mouse cortex [36], whereas in human frontal cortex layer 3 neurons contain the greatest number of dendritic spines [37], [38]. The enrichment of PSD proteins and their increased expression within layer 5 of both mouse and macaque cortex may be necessary for distinctive functions of layer 5 neurons. Future work examining the activity of layer 5 neurons in the absence of layer 5-specific PSD proteins could provide important insights into the functional role of these proteins within this layer. In addition, whether this increased expression of PSD proteins within layer 5 holds true for human cortex will be enlightening. Recent work has shown a high correlation between human and mouse layer expression of a subset of genes (∼1000) [39]. Only four genes overlap between the human PSD dataset and the genes profiled in human cortical layers. Two of these genes are expressed in layer 5 of the human visual or temporal cortex, but none of these genes has expression unique or enriched in layer 5 (Table S5). Future genome-wide layer expression data in human brain should more fully address this question.

In addition to the unique properties of layer 5, the cortical laminae can be roughly divided into two classes based on the projections of neurons within the layers. The neurons of upper layers (layers 2–3) are the main source of intracortical connections (at least in primates), while neurons of the deep layers, layers 5 and 6, primarily project to the “older” subcortical areas of the brain with layer 6 neurons projecting to the thalamus and layer 5 neurons projecting to the midbrain, hindbrain, and spinal cord [35], [40], [41]. Based on these different circuits, a simplified model can be proposed in which the deep layers disseminate the output of the information assessed by the superficial layers [42]. Therefore, the additional evolutionary constraints on PSD genes in deeper layers may have been necessary for the development of an organized cortex and integration with subcortical areas, whereas fewer evolutionary constraints on PSD genes in the upper layers of the cortex may have facilitated the evolution of a six-layered cortex and the emergence of higher cognitive functions through cortico-cortical connectivity. We need to be cautious about the interpretation and extension of our findings to human brain, since our data combine PSD data from human brain tissue with layer specific gene expression patterns in mouse somatosensory cortex or rhesus macaque visual cortex. Layer thickness throughout the cortex, number of cortical areas, laminar projections among cortical areas, and areal boundaries can also vary considerably among mammalian species [41], [43]–[45]; therefore, comparisons across different regions need to be interpreted carefully. For example, comparisons of prefrontal cortex between human and mouse brain may not be applicable, as there is debate as to whether rodents even have a prefrontal cortex [46]. Here, we show data from sublayers of layers 4 and 5 from the mouse somatosensory cortex data and sublayers of layer 4 in the rhesus macaque visual cortex data. In addition, neither of these brain regions have a prominent layer 5 with distinct Betz cells as is seen in motor cortex [47]. Thus, it is unclear as to whether our layer 5 results would also apply to motor cortex. The confirmation of our findings in macaque brain is important though, especially as the laminar distribution of a number of genes is highly conserved between human and macaque but not between human and mouse visual cortex [22]. However, human cortex does have different lamination patterns even from macaque [48]. Finally, when whole tissue pieces have been utilized for gene expression profiling in human cortex, there are very few genes that distinguish the cortical regions in adult human brain [22], [49], [50], supporting our use of multiple cortical areas for these analyses but highlighting the need for higher resolution data. Human in situ hybridization data are available through the Allen Brain Institute (http://www.brain-map.org/), and a recent study from the ABI has correlated expression of approximately 1000 genes in the visual or temporal cortex finding roughly an 80% similarity rate between human and mouse cortical layer expression [39]. However, future work examining gene expression at a quantitative level in individual layers of the human brain should provide insight into how much the data presented here can be extended to the human cortex.

## Materials and Methods

### Genome Annotation and Evolutionary Rate Calculations

The genome annotation information of mouse was downloaded from the Ensembl database (http://www.ensembl.org/); Ensembl genes version 64 was used. Genes for presynaptic membrane proteins were obtained from (http://www.informatics.jax.org/searches/GOannot_report.cgi?id=GO:0042734). To measure the evolutionary rate of these genes, the human genome was used as the reference genome. Orthologous gene pairs between mouse and human, including the synonymous substitution rate (dS) and non-synonymous substitution rate (dN) were obtained from Ensembl using the BioMart batch query tool. The synonymous substitution rate (dS) and non-synonymous substitution rate (dN) between orthologous pairs were calculated by codeml in PAML [51]. The ratio of dN and dS (dN/dS) was used to measure the evolutionary rate of mouse genes in this study.

### Expression Analyses

Cortical layer-enriched gene expression in mouse was collected from a transcriptomic atlas of mouse somatosensory cortex [8]. Layer enrichment probability >0.5 (uncalib) was set as the cutoff of layer enriched genes for each layer. To investigate whether the expression level in cortex plays an important role in the evolution of PSD genes (total PSD genes were used in all of the studies [5]), the transcriptional levels of each gene from each layer were downloaded from the supplementary web resources (http://wwwfgu.anat.ox.ac.uk/~grantb/mouse_layers/; the data in combined_fpkms.tsv were used). If there were two samples from the same layer, the average expression of these two samples was used to represent the expression abundance of genes in this layer in the statistical comparisons. Note that the mouse expression data used here is limited to the somatosensory cortex. The mouse brain proteomics and mouse brain plasma membrane proteomics were from HPO [52]. Mouse PSD genes were reported as before [14]. Upon comparison of PSD genes and other brain related genes in each dataset, all non-PSD genes were included from each dataset. To control for the effect of expression level in cortex, the residues from a loess regression model were used to represent the evolutionary rate of mouse proteins. All of the statistical analyses were performed in R.

### Rhesus Macaque Neocortex Transcriptome Data

Rhesus macaque neocortex transcriptome data were obtained from a recently published resource [22]. Data from primary visual cortex (V1) and secondary visual cortex (V2) were used (well id from 11416 to 11402) since these datasets contained the most detailed layer expression data. The mean expression profile of each gene was used to represent the transcriptional level of that gene. Next, the average expression of each gene in both deep layers (layers 5–6) and upper layers (layer 1–3) were calculated.

### Co-expression Network and Transcription Factor Correlations

To construct the co-expression network, we calculated the Pearson correlation values for each gene pair in twenty large datasets, which in total contains 539 arrays from different mouse brain areas [27]. Genome-wide mouse transcription factors predicted from hidden Markov models were obtained from a transcription factor prediction database (DBD) [29]. We mapped both the layer-enriched PSD genes and the transcription factors to the co-expression data. 439 transcription factors are included in the final dataset. To examine whether higher expression profiles of PSD genes in deeper layers are connected to layer-enriched transcription factors, we first compared both the co-expression between PSD gene pairs and between PSD genes and layer enriched transcriptional factors with 100 randomly sampled genes as a control. Next, we compared the co-expression of PSD gene pairs and of PSD genes and layer-enriched transcriptional factors in each layer. All the comparisons are by Wilcoxon rank sum test. In total 92,352 TF- PSD gene pairs and 44,477 PSD-PSD gene pairs were used in this comparison.

## Supporting Information

Figure S1 Comparison of the expression level of PSD genes in different layers of the rhesus macaque primary visual cortex (V1) (top) and secondary visual cortex (V2) (bottom).
(TIFF)

Figure S2 **Density plot of PSD genes in each layer using mouse gene expression data.**
(TIFF)

Figure S3 Density plot of PSD genes in each layer using macaque gene expression data from A) primary visual cortex (V1) and B) secondary visual cortex (V2).
(TIFF)

Table S1 Expression levels of genes encoding for presynaptic membrane proteins in deep layers have higher expression levels compared to those genes in upper layers.
(DOCX)

Table S2 After normalization to *Aldh1l1*, a highly specific astrocyte marker to control for the cellular composition, PSD genes in deep layers still show higher expression levels compared to upper layers.
(DOCX)

Table S3 Co-expression between PSD gene pairs when using the top 50% highly expressed or bottom 50% expressed PSD genes to avoid the influence of expression level on co-expression analyses.
(DOCX)

Table S4 Co-expression between PSD genes and transcription factors when using the top 50% highly expressed or bottom 50% expressed PSD genes to avoid the influence of expression level on co-expression analyses.
(DOCX)

Table S5 Expression values of layer related PSD genes in visual and temporal cortices of adult human brains.
(DOCX)
