# Supplementary figures and images for: Differential Functional Constraints on the Evolution of Postsynaptic Density Proteins in Neocortical Laminae
Source: PLoS One. 2012 Jun 28;7(6):e39686. doi: 10.1371/journal.pone.0039686 (PMC3386249; doi:10.1371/journal.pone.0039686)

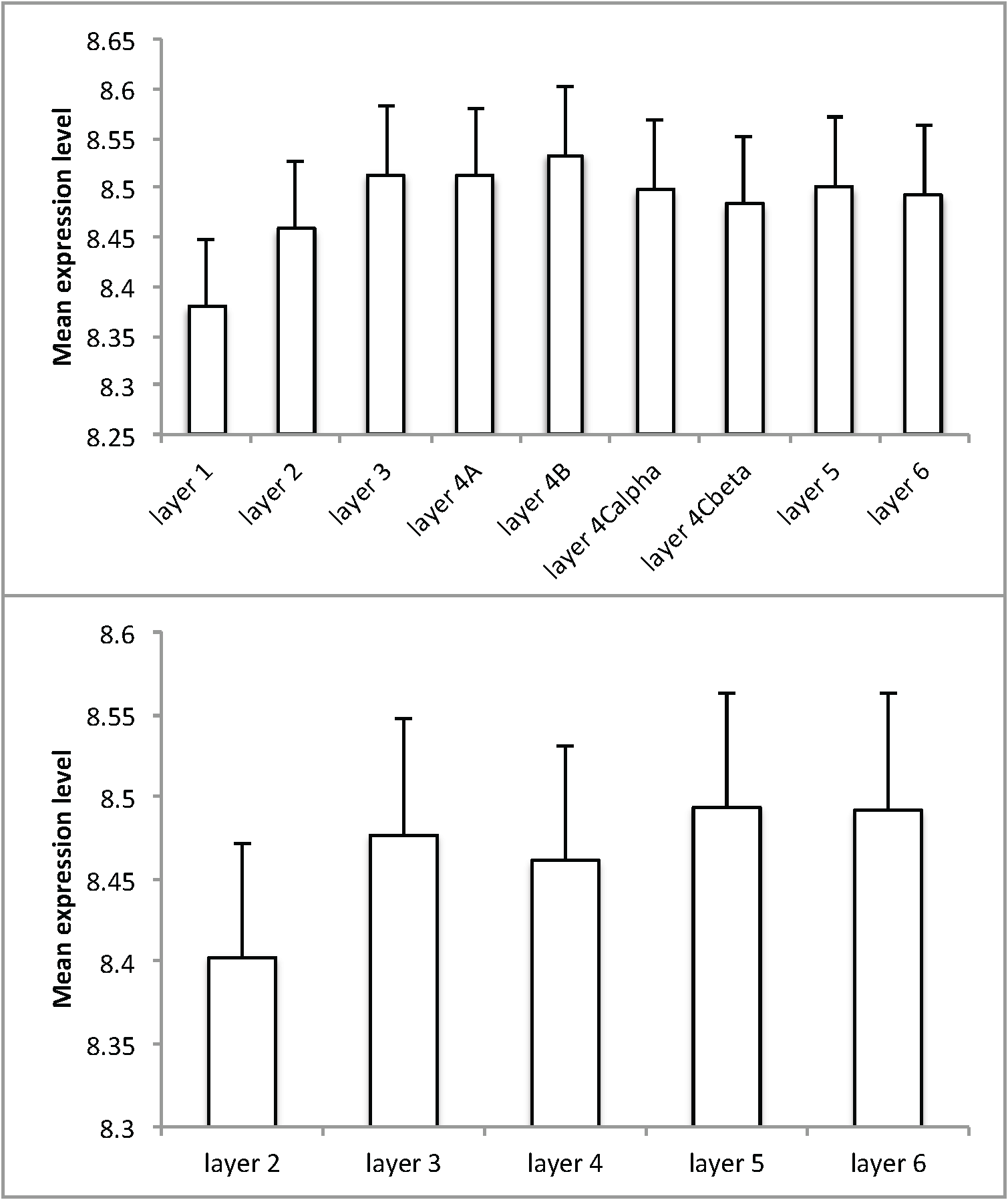

Supplement: Figure S1 — Comparison of the expression level of PSD genes in different layers of the rhesus macaque primary visual cortex (V1) (top) and secondary visual cortex (V2) (bottom). (TIFF) [file pone.0039686.s001.tiff]

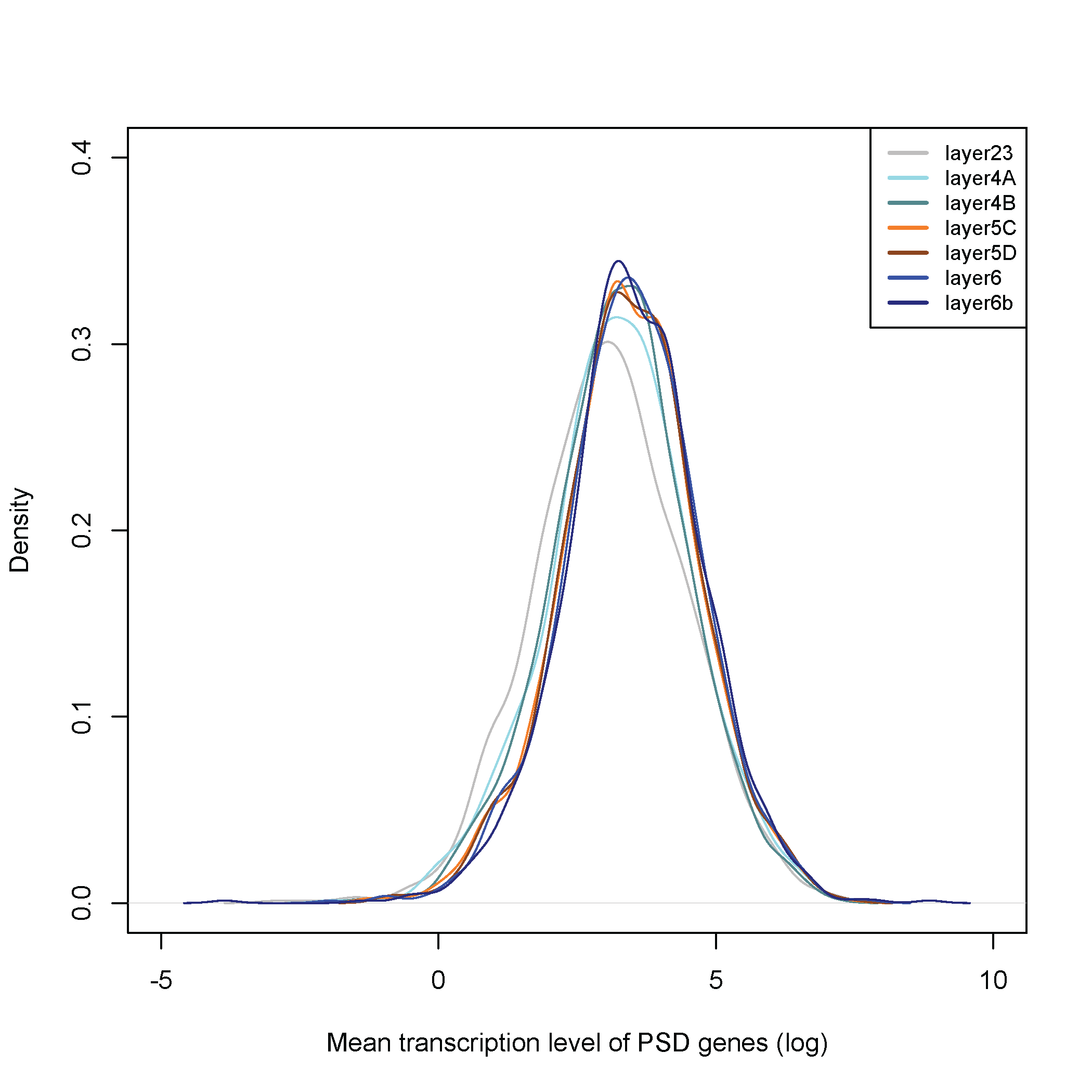

Supplement: Figure S2 — Density plot of PSD genes in each layer using mouse gene expression data. (TIFF) [file pone.0039686.s002.tiff]

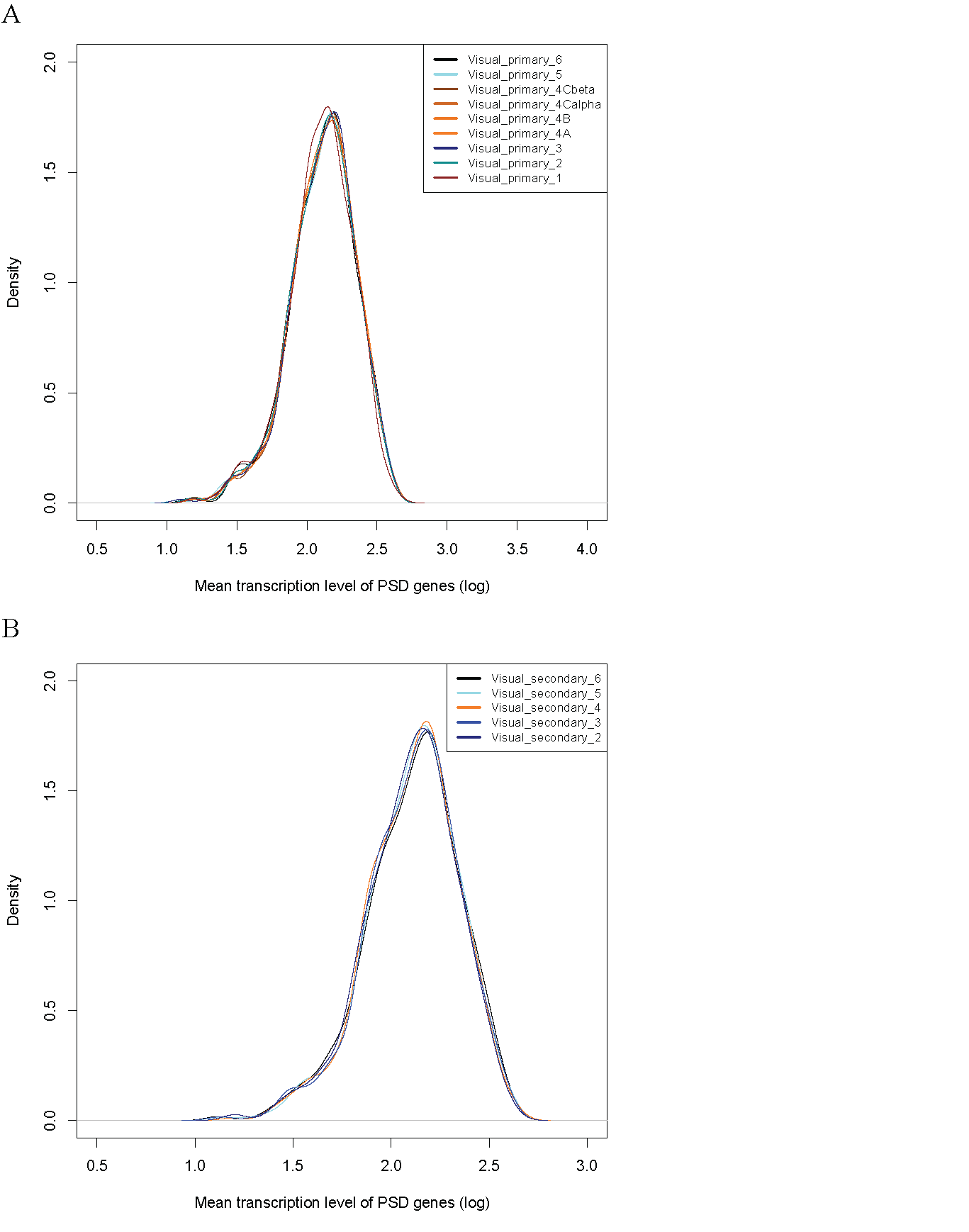

Supplement: Figure S3 — Density plot of PSD genes in each layer using macaque gene expression data from A) primary visual cortex (V1) and B) secondary visual cortex (V2). (TIFF) [file pone.0039686.s003.tiff]
