# Supplementary material for: Differential Functional Constraints on the Evolution of Postsynaptic Density Proteins in Neocortical Laminae
Source: PLoS One. 2012 Jun 28;7(6):e39686. doi: 10.1371/journal.pone.0039686 (PMC3386249; doi:10.1371/journal.pone.0039686)
Supplement: Table S1 — Expression levels of genes encoding for presynaptic membrane proteins in deep layers have higher expression levels compared to those genes in upper layers. (DOCX) [file pone.0039686.s004.docx]

Table S1. Expression levels of genes encoding for presynaptic membrane proteins in deep layers have higher expression levels compared to those genes in upper layers.

|  | Mean expression level | Standard deviation |
| --- | --- | --- |
| Layer 2/3 | 69.81 | 20.96 |
| Layer 4A | 85.98 | 22.61 |
| Layer 4B | 80.71 | 21.05 |
| Layer 5C | 96.99 | 29.88 |
| Layer 5D | 95.03 | 28.49 |
| Layer 6 | 99.71 | 28.55 |
| Layer 6b | 96.05 | 27.51 |
