# Supplementary material for: Differential Functional Constraints on the Evolution of Postsynaptic Density Proteins in Neocortical Laminae
Source: PLoS One. 2012 Jun 28;7(6):e39686. doi: 10.1371/journal.pone.0039686 (PMC3386249; doi:10.1371/journal.pone.0039686)
Supplement: Table S2 — After normalization to Aldh1l1, a highly specific astrocyte marker to control for the cellular composition, PSD genes in deep layers still show higher expression levels compared to upper layers. (DOCX) [file pone.0039686.s005.docx]

Table S2. After normalization to *Aldh1l1*, a highly specific astrocyte marker to control for the cellular composition, PSD genes in deep layers still show higher expression levels compared to upper layers.

|  | Normalized expression | Standard deviation | Expression of *Aldh1l1* |
| --- | --- | --- | --- |
| Layer 2/3 | 2.15 | 0.12 | 24.65 |
| Layer 4A | 1.80 | 0.09 | 32.67 |
| Layer 4B | 2.48 | 0.12 | 22.45 |
| Layer 5C | 2.45 | 0.12 | 26.58 |
| Layer 5D | 3.24 | 0.16 | 20.46 |
| Layer 6 | 3.91 | 0.21 | 17.56 |
| Layer 6b | 3.65 | 0.32 | 20.98 |
