# Supplementary material for: Differential Functional Constraints on the Evolution of Postsynaptic Density Proteins in Neocortical Laminae
Source: PLoS One. 2012 Jun 28;7(6):e39686. doi: 10.1371/journal.pone.0039686 (PMC3386249; doi:10.1371/journal.pone.0039686)
Supplement: Table S3 — Co-expression between PSD gene pairs when using the top 50% highly expressed or bottom 50% expressed PSD genes to avoid the influence of expression level on co-expression analyses. (DOCX) [file pone.0039686.s006.docx]

Table S3. Co-expression between PSD gene pairs when using the top 50% highly expressed or bottom 50% expressed PSD genes to avoid the influence of expression level on co-expression analyses.

| Top 50% | Mean co-expression level | Standard deviation | Sample number |
| --- | --- | --- | --- |
| Layer 6 | 0.516 | 0.037 | 91 |
| Layer 5 | 0.378 | 0.003 | 9730 |
| Layer 4 | 0.271 | 0.092 | 10 |
| Layer 2/3 | 0.254 | 0.007 | 2556 |
| Layer 6 and layer 2/3 | | p-value = 1.7 × 10^-13^ | |
| Layer 6 and layer 4 | | p-value = 6 × 10^-3^ | |
| Layer 5 and layer 2/3 | | p-value < 2.2 × 10^-16^ | |
| Layer 5 and layer 4 | | p-value = 1.9 × 10^-1^ | |

| Bottom 50% | Mean co-expression level | Standard deviation | Sample number |
| --- | --- | --- | --- |
| Layer 6 | 0.259 | 0.032 | 105 |
| Layer 5 | 0.254 | 0.005 | 7140 |
| Layer 4 | 0.043 | 0.080 | 15 |
| Layer 2/3 | 0.112 | 0.009 | 1225 |
| Layer 6 and layer 2/3 | | p-value = 4 × 10^-5^ | |
| Layer 6 and layer 4 | | p-value = 2.5 × 10^-2^ | |
| Layer 5 and layer 2/3 | | p-value < 2.2 × 10^-16^ | |
| Layer 5 and layer 4 | | p-value = 2.8 × 10^-2^ | |
