# Supplementary material for: Differential Functional Constraints on the Evolution of Postsynaptic Density Proteins in Neocortical Laminae
Source: PLoS One. 2012 Jun 28;7(6):e39686. doi: 10.1371/journal.pone.0039686 (PMC3386249; doi:10.1371/journal.pone.0039686)
Supplement: Table S4 — Co-expression between PSD genes and transcription factors when using the top 50% highly expressed or bottom 50% expressed PSD genes to avoid the influence of expression level on co-expression analyses. (DOCX) [file pone.0039686.s007.docx]

Table S4. Co-expression between PSD genes and transcription factors when using the top 50% highly expressed or bottom 50% expressed PSD genes to avoid the influence of expression level on co-expression analyses.

| Top 50% | Mean co-expression level | Standard deviation | Sample number |
| --- | --- | --- | --- |
| Layer 6 | 0.165 | 0.005 | 4848 |
| Layer 5 | 0.152 | 0.003 | 12524 |
| Layer 4 | 0.150 | 0.007 | 3636 |
| Layer 2/3 | 0.108 | 0.002 | 23836 |
| Layer 6 and layer 2/3 | | p-value = 1.1 × 10^-1^ | |
| Layer 6 and layer 4 | | p-value < 2.2 × 10^-16^ | |
| Layer 5 and layer 2/3 | | p-value < 2.2 × 10^-16^ | |
| Layer 5 and layer 4 | | p-value = 4.9 × 10^-2^ | |

| Bottom 50% | Mean co-expression level | Standard deviation | Sample number |
| --- | --- | --- | --- |
| Layer 6 | 0.149 | 0.006 | 4044 |
| Layer 5 | 0.134 | 0.003 | 10447 |
| Layer 4 | 0.117 | 0.007 | 3033 |
| Layer 2/3 | 0.105 | 0.002 | 19883 |
| Layer 6 and layer 2/3 | | p-value < 2.2 × 10^-16^ | |
| Layer 6 and layer 4 | | p-value = 4.6 × 10^-4^ | |
| Layer 5 and layer 2/3 | | p-value = 7 × 10^-11^ | |
| Layer 5 and layer 4 | | p-value = 4.8 × 10^-1^ | |
