# Supplementary material for: Differential Functional Constraints on the Evolution of Postsynaptic Density Proteins in Neocortical Laminae
Source: PLoS One. 2012 Jun 28;7(6):e39686. doi: 10.1371/journal.pone.0039686 (PMC3386249; doi:10.1371/journal.pone.0039686)
Supplement: Table S5 — Expression values of layer related PSD genes in visual and temporal cortices of adult human brains. (DOCX) [file pone.0039686.s008.docx]

Table S5. Expression values of layer related PSD genes in visual and temporal cortices of adult human brains.

| Gene symbol | Cortical layer | Expression V1 | Expression V2 | Expression Temporal |
| --- | --- | --- | --- | --- |
| *WFS1* | Layer 2 | 3 | 3 | 3 |
| *CRYM* | Layer 2/3/5/6 | 5 | 5 | 5 |
| *ATP2B4* | Layer 2/3/6 | 3 | 3 | N/A |
| *PDE1A* | Layer 5/6 | 4 | 3 | 3 |

Data are from Zeng, H. et al., 2012, *Cell*. Expression levels are from *in situ* hybridization data and therefore are not quantitative. Values are scored from 1-5, with 5 being the greatest amount of expression. Layer information denotes which layers have at least some expression.
